# Supplementary material for: SARS-CoV-2 suppression and early closure of bars and restaurants: a longitudinal natural experiment
Source: Sci Rep. 2022 Jul 23;12:12623. doi: 10.1038/s41598-022-16428-4 (PMC9308477; doi:10.1038/s41598-022-16428-4)
Supplement: Supplementary file 1 — Supplementary Information. [file 41598_2022_16428_MOESM1_ESM.pdf]

Supplementary Information Appendix

Table SI1: Descriptive Statistics

	Full Sample	Prefectures with the SE	Prefectures without the SE
	(1)	(2)	(3)
	Mean	Mean	Mean
	(S.D.)	(S.D.)	(S.D.)
**Dependent Variables**			
Utilization of Restaurants and Bars During Past One Month	0.378	0.387	0.351
	(0.485)	(0.487)	(0.477)
Fever	0.0184	0.0192	0.0161
	(0.135)	(0.137)	(0.126)
Sore Throat	0.114	0.114	0.112
	(0.317)	(0.318)	(0.316)
Cough	0.131	0.129	0.136
	(0.337)	(0.335)	(0.343)
Headache	0.0187	0.0198	0.0155
	(0.135)	(0.139)	(0.123)
Smell and Taste Disorder	0.0122	0.0125	0.0114
	(0.110)	(0.111)	(0.106)
**Individual Characteristics**			
Age	50.53	49.92	52.34
	(16.510)	(16.600)	(16.100)
College Graduate	0.512	0.531	0.455
	(0.500)	(0.499)	(0.498)
Married	0.629	0.615	0.67
	(0.483)	(0.487)	(0.470)
Worker	0.642	0.648	0.622
	(0.480)	(0.478)	(0.485)
Household with Income More Than JPY 7 Million	0.325	0.338	0.285
	(0.468)	(0.473)	(0.452)
Smoker	0.21	0.212	0.205
	(0.407)	(0.408)	(0.403)
Used Japanese Pubs and Bars During the Period Before the Pandemic	0.456	0.474	0.401
(“Japanese Pub Users”)	(0.498)	(0.499)	(0.490)
Not Scared at SARS-CoV-2	0.373	0.379	0.356
	(0.484)	(0.485)	(0.479)
Observations	25,338	19,007	6,331

Notes: The sample consists of persons who lived within 50 Km from the border which separates the prefectures with and without the SE (state of emergency) declaration.

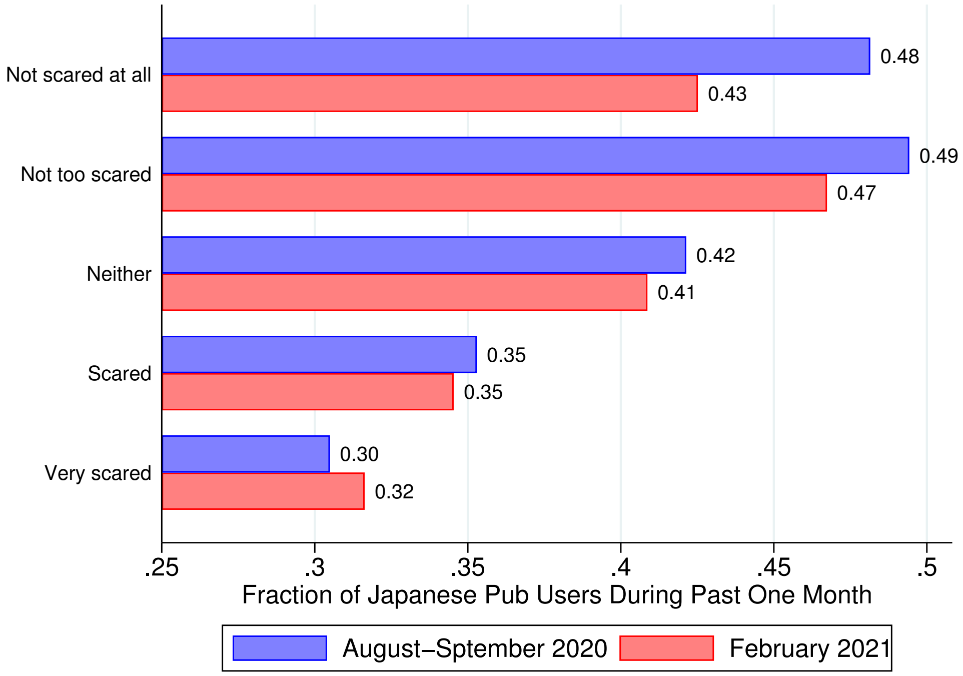

Figure SI1: Utilization Rate of Japanese Pubs and Bars by the Extent of Fear of SARS-CoV-2

Table SI2: Effects on the Utilization of Japanese Pubs and Bars among Young Persons

	(1)	(2)	(3)	(4)	(5)
Panel A. Fixed Effect Linear Probability Model					
Treat	-0.034***	-0.048***	-0.052**	-0.072***	-0.072**
	(-0.058 - -0.009)	(-0.077 - -0.018)	(-0.093 - -0.010)	(-0.122 - -0.022)	(-0.139 - -0.005)
Panel B. Random Effect Logistic Regression Model					
Treat	0.830**	0.765***	0.766**	0.688**	0.654**
	(0.697 - 0.988)	(0.629 - 0.931)	(0.592 - 0.992)	(0.515 - 0.918)	(0.465 - 0.921)
Prefecture FEs for Logistic Regression	yes	yes	yes	yes	yes
Individual Level Covariates	no	yes	yes	yes	yes
Exclude Respondents far from 50 Km from the Border	no	no	yes	yes	yes
Exclude Respondents within 10 Km from the Border	no	no	no	yes	no
Exclude Respondents within 20 Km from the Border	no	no	no	no	yes
Observations	12,008	9,573	7,580	6,556	4,609

Notes: The dependent variable is a binary variable for the utilization of full service restaurants bars during past one month. Panel A summarizes the results from fixed effects linear probability regression model and Panel B summarizes the results from random effect logistic regression model. Coefficients and 95 confidence intervals are reported in Panel A and odds ratios and 95 confidence intervals are reported in Panel B. Standard errors clustered in 47 prefectures are reported in parentheses in Panel A. *** p<0.01; ** p<0.05; * p<0.1.

Table SI3: Effects on the Utilization of Japanese Pubs and Bars among Elderly Persons

	(1)	(2)	(3)	(4)	(5)
Panel A. Fixed Effect Linear Probability Model					
Treat	-0.008	-0.006	-0.010	-0.014	-0.026*
	(-0.025 - 0.009)	(-0.026 - 0.015)	(-0.033 - 0.014)	(-0.042 - 0.014)	(-0.054 - 0.002)
Panel B. Random Effect Logistic Regression Model					
Treat	0.949	0.957	0.938	0.908	0.858
	(0.845 - 1.065)	(0.840 - 1.090)	(0.792 - 1.110)	(0.752 - 1.097)	(0.686 - 1.075)
Prefecture FEs for Logistic Regression	yes	yes	yes	yes	yes
Individual Level Covariates	no	yes	yes	yes	yes
Exclude Respondents far from 50 Km from the Border	no	no	yes	yes	yes
Exclude Respondents within 10 Km from the Border	no	no	no	yes	no
Exclude Respondents within 20 Km from the Border	no	no	no	no	yes
Observations	29,230	23,290	17,758	15,022	10,628

Notes: The dependent variable is a binary variable for the utilization of full service restaurants bars during past one month. Panel A summarizes the results from fixed effects linear probability regression model and Panel B summarizes the results from random effect logistic regression model. Coefficients and 95 confidence intervals are reported in Panel A and odds ratios and 95 confidence intervals are reported in Panel B. Standard errors clustered in 47 prefectures are reported in parentheses in Panel A. *** p<0.01; ** p<0.05; * p<0.1.

Table SI4: Effects on the Utilization of Japanese Pubs and Bars among College Graduates

	(1)	(2)	(3)	(4)	(5)
Panel A. Fixed Effect Linear Probability Model					
Treat	-0.031***	-0.037***	-0.033*	-0.048**	-0.046*
	(-0.053 - -0.009)	(-0.063 - -0.011)	(-0.068 - 0.002)	(-0.089 - -0.007)	(-0.093 - 0.001)
Panel B. Random Effect Logistic Regression Model					
Treat	0.832**	0.800***	0.844*	0.760**	0.789*
	(0.722 - 0.957)	(0.685 - 0.935)	(0.691 - 1.031)	(0.608 - 0.951)	(0.608 - 1.024)
Prefecture FEs for Logistic Regression	yes	yes	yes	yes	yes
Individual Level Covariates	no	yes	yes	yes	yes
Exclude Respondents far from 50 Km from the Border	no	no	yes	yes	yes
Exclude Respondents within 10 Km from the Border	no	no	no	yes	no
Exclude Respondents within 20 Km from the Border	no	no	no	no	yes
Observations	20,017	16,581	13,427	11,630	8,452

Notes: The dependent variable is a binary variable for the utilization of full service restaurants bars during past one month. Panel A summarizes the results from fixed effects linear probability regression model and Panel B summarizes the results from random effect logistic regression model. Coefficients and 95 confidence intervals are reported in Panel A and odds ratios and 95 confidence intervals are reported in Panel B. Standard errors clustered in 47 prefectures are reported in parentheses in Panel A. *** $p<0.01$; ** $p<0.05$; * $p<0.1$.

Table SI5: Effects on the Utilization of Japanese Pubs and Bars among Non College Graduates

	(1)	(2)	(3)	(4)	(5)
Panel A. Fixed Effect Linear Probability Model					
Treat	-0.009	-0.005	-0.013	-0.019	-0.039*
	(-0.030 - 0.013)	(-0.029 - 0.019)	(-0.044 - 0.017)	(-0.055 - 0.018)	(-0.079 - 0.002)
Panel B. Random Effect Logistic Regression Model					
Treat	0.951	0.970	0.907	0.890	0.766*
	(0.832 - 1.088)	(0.832 - 1.131)	(0.744 - 1.107)	(0.712 - 1.113)	(0.584 - 1.004)
Prefecture FEs for Logistic Regression	yes	yes	yes	yes	yes
Individual Level Covariates	no	yes	yes	yes	yes
Exclude Respondents far from 50 Km from the Border	no	no	yes	yes	yes
Exclude Respondents within 10 Km from the Border	no	no	no	yes	no
Exclude Respondents within 20 Km from the Border	no	no	no	no	yes
Observations	21,131	16,229	11,876	9,914	6,755

Notes: The dependent variable is a binary variable for the utilization of full service restaurants bars during past one month. Panel A summarizes the results from fixed effects linear probability regression model and Panel B summarizes the results from random effect logistic regression model. Coefficients and 95 confidence intervals are reported in Panel A and odds ratios and 95 confidence intervals are reported in Panel B. Standard errors clustered in 47 prefectures are reported in parentheses in Panel A. *** $p<0.01$; ** $p<0.05$; * $p<0.1$.

Table SI6: Effects on the Utilization of Japanese Pubs and Bars among Japanese Pub Users

	(1)	(2)	(3)	(4)	(5)
Panel A. Fixed Effect Linear Probability Model					
Treat	-0.016	-0.023	-0.027	-0.054**	-0.053*
	(-0.043 - 0.012)	(-0.052 - 0.006)	(-0.069 - 0.015)	(-0.096 - -0.013)	(-0.109 - 0.003)
Panel B. Random Effect Logistic Regression Model					
Treat	0.914	0.882	0.883	0.754**	0.756*
	(0.788 - 1.060)	(0.751 - 1.037)	(0.714 - 1.093)	(0.594 - 0.957)	(0.570 - 1.002)
Prefecture FEs for Logistic Regression	yes	yes	yes	yes	yes
Individual Level Covariates	no	yes	yes	yes	yes
Exclude Respondents far from 50 Km from the Border	no	no	yes	yes	yes
Exclude Respondents within 10 Km from the Border	no	no	no	yes	no
Exclude Respondents within 20 Km from the Border	no	no	no	no	yes
Observations	17,282	14,697	11,550	9,972	7,124

Notes: The dependent variable is a binary variable for the utilization of full service restaurants bars during past one month. Panel A summarizes the results from fixed effects linear probability regression model and Panel B summarizes the results from random effect logistic regression model. Coefficients and 95 confidence intervals are reported in Panel A and odds ratios and 95 confidence intervals are reported in Panel B. Standard errors clustered in 47 prefectures are reported in parentheses in Panel A. *** $p<0.01$; ** $p<0.05$; * $p<0.1$.

Table SI7: Effects on the Utilization of Japanese Pubs and Bars among Non Japanese Pub Users

	(1)	(2)	(3)	(4)	(5)
Panel A. Fixed Effect Linear Probability Model					
Treat	-0.013*	-0.010	-0.013	-0.009	-0.027*
	(-0.028 - 0.002)	(-0.029 - 0.008)	(-0.033 - 0.007)	(-0.036 - 0.018)	(-0.055 - 0.002)
Panel B. Random Effect Logistic Regression Model					
Treat	0.925	0.921	0.909	0.933	0.841
	(0.815 - 1.050)	(0.796 - 1.067)	(0.754 - 1.096)	(0.757 - 1.151)	(0.655 - 1.080)
Prefecture FEs for Logistic Regression	yes	yes	yes	yes	yes
Individual Level Covariates	no	yes	yes	yes	yes
Exclude Respondents far from 50 Km from the Border	no	no	yes	yes	yes
Exclude Respondents within 10 Km from the Border	no	no	no	yes	no
Exclude Respondents within 20 Km from the Border	no	no	no	no	yes
Observations	23,956	18,166	13,788	11,606	8,113

Notes: The dependent variable is a binary variable for the utilization of full service restaurants bars during past one month. Panel A summarizes the results from fixed effects linear probability regression model and Panel B summarizes the results from random effect logistic regression model. Coefficients and 95 confidence intervals are reported in Panel A and odds ratios and 95 confidence intervals are reported in Panel B. Standard errors clustered in 47 prefectures are reported in parentheses in Panel A. *** $p<0.01$; ** $p<0.05$; * $p<0.1$.

Table SI8: Effects on the Utilization of Japanese Pubs and Bars among ”Not Scared”

	(1)	(2)	(3)	(4)	(5)
Panel A. Fixed Effect Linear Probability Model					
Treat	-0.024	-0.028*	-0.030	-0.047**	-0.063**
	(-0.053 - 0.005)	(-0.059 - 0.002)	(-0.068 - 0.009)	(-0.093 - -0.001)	(-0.118 - -0.007)
Panel B. Random Effect Logistic Regression Model					
Treat	0.870*	0.843*	0.838	0.765**	0.674**
	(0.741 - 1.022)	(0.703 - 1.010)	(0.663 - 1.060)	(0.587 - 0.997)	(0.493 - 0.923)
Prefecture FEs for Logistic Regression	yes	yes	yes	yes	yes
Individual Level Covariates	no	yes	yes	yes	yes
Exclude Respondents far from 50 Km from the Border	no	no	yes	yes	yes
Exclude Respondents within 10 Km from the Border	no	no	no	yes	no
Exclude Respondents within 20 Km from the Border	no	no	no	no	yes
Observations	14,876	12,017	9,461	8,047	5,730

Notes: The dependent variable is a binary variable for the utilization of full service restaurants bars during past one month. Panel A summarizes the results from fixed effects linear probability regression model and Panel B summarizes the results from random effect logistic regression model. Coefficients and 95 confidence intervals are reported in Panel A and odds ratios and 95 confidence intervals are reported in Panel B. Standard errors clustered in 47 prefectures are reported in parentheses in Panel A. *** p<0.01; ** p<0.05; * p<0.1.

Table SI9: Effects on the Utilization of Japanese Pubs and Bars among "Scared"

	(1)	(2)	(3)	(4)	(5)
Panel A. Fixed Effect Linear Probability Model					
Treat	-0.015	-0.015	-0.020*	-0.025**	-0.032**
	(-0.033 - 0.004)	(-0.037 - 0.007)	(-0.045 - 0.004)	(-0.051 - -0.000)	(-0.063 - -0.001)
Panel B. Random Effect Logistic Regression Model					
Treat	0.915	0.906	0.888	0.853	0.833
	(0.812 - 1.032)	(0.791 - 1.037)	(0.745 - 1.059)	(0.701 - 1.037)	(0.660 - 1.051)
Prefecture FEs for Logistic Regression	yes	yes	yes	yes	yes
Individual Level Covariates	no	yes	yes	yes	yes
Exclude Respondents far from 50 Km from the Border	no	no	yes	yes	yes
Exclude Respondents within 10 Km from the Border	no	no	no	yes	no
Exclude Respondents within 20 Km from the Border	no	no	no	no	yes
Observations	26,362	20,846	15,877	13,531	9,507

Notes: The dependent variable is a binary variable for the utilization of full service restaurants bars during past one month. Panel A summarizes the results from fixed effects linear probability regression model and Panel B summarizes the results from random effect logistic regression model. Coefficients and 95 confidence intervals are reported in Panel A and odds ratios and 95 confidence intervals are reported in Panel B. Standard errors clustered in 47 prefectures are reported in parentheses in Panel A. *** $p<0.01$; ** $p<0.05$; * $p<0.1$.

Table SI10: Effects on Symptoms Like SARS-CoV-2 among Young Persons

	(1)	(2)	(3)	(4)	(5)
Panel A. Fixed Effect Linear Probability Model					
High Fever	0.002	-0.002	-0.004	-0.002	-0.001
	(-0.007 - 0.011)	(-0.013 - 0.009)	(-0.020 - 0.013)	(-0.021 - 0.018)	(-0.027 - 0.025)
Sore Throat	0.005	0.003	-0.015	-0.019	-0.040
	(-0.023 - 0.033)	(-0.032 - 0.039)	(-0.050 - 0.021)	(-0.062 - 0.024)	(-0.094 - 0.014)
Cough	0.007	0.006	0.004	-0.012	-0.030
	(-0.014 - 0.028)	(-0.019 - 0.031)	(-0.028 - 0.036)	(-0.046 - 0.022)	(-0.078 - 0.018)
Headache	-0.006	-0.011	-0.001	-0.001	-0.003
	(-0.020 - 0.007)	(-0.025 - 0.004)	(-0.022 - 0.020)	(-0.024 - 0.022)	(-0.026 - 0.021)
Smell and Taste Disorder	0.002	0.000	-0.005	-0.000	-0.006
	(-0.007 - 0.012)	(-0.010 - 0.010)	(-0.016 - 0.006)	(-0.012 - 0.011)	(-0.019 - 0.007)
Panel B. Random Effect Logistic Regression Model					
High Fever	1.097	0.997	0.909	0.960	0.829
	(0.675 - 1.784)	(0.598 - 1.663)	(0.451 - 1.831)	(0.431 - 2.134)	(0.329 - 2.086)
Sore Throat	1.068	1.066	0.908	0.920	0.742
	(0.840 - 1.357)	(0.820 - 1.387)	(0.637 - 1.295)	(0.623 - 1.359)	(0.465 - 1.184)
Cough	1.134	1.093	1.096	0.936	0.729
	(0.867 - 1.483)	(0.813 - 1.468)	(0.743 - 1.616)	(0.610 - 1.436)	(0.433 - 1.228)
Headache	0.783	0.700	1.118	1.291	1.084
	(0.487 - 1.259)	(0.421 - 1.165)	(0.563 - 2.221)	(0.595 - 2.802)	(0.428 - 2.748)
Smell and Taste Disorder	1.141	1.128	0.723	1.062	0.754
	(0.582 - 2.235)	(0.546 - 2.332)	(0.252 - 2.075)	(0.344 - 3.277)	(0.212 - 2.689)
Prefecture FEs for Logistic Regression	yes	yes	yes	yes	yes
Individual Level Covariates	no	yes	yes	yes	yes
Exclude Respondents far from 50 Km from the Border	no	no	yes	yes	yes
Exclude Respondents within 10 Km from the Border	no	no	no	yes	no
Exclude Respondents within 20 Km from the Border	no	no	no	no	yes
Observations	12,008	9,573	7,580	6,556	4,609

Notes: The dependent variable is a binary variable for 5 subjective symptom like SARS-CoV-2 (high fever, sore throat, cough, headache, and smell and taste disorder). Panel A summarizes the results from fixed effects linear probability regression model and Panel B summarizes the results from random effect logistic regression model. Coefficients and 95 confidence intervals are reported in Panel A and odds ratios and 95 confidence intervals are reported in Panel B. Standard errors clustered in 47 prefectures are reported in parentheses in Panel A. *** p<0.01; ** p<0.05; * p<0.1.

Table SI11: Effects on Symptoms Like SARS-CoV-2 among Elderly Persons

	(1)	(2)	(3)	(4)	(5)
Panel A. Fixed Effect Linear Probability Model					
High Fever	0.004*	0.005**	0.004	0.005	0.007
	(-0.000 - 0.008)	(0.000 - 0.010)	(-0.003 - 0.012)	(-0.005 - 0.015)	(-0.005 - 0.018)
Sore Throat	-0.000	0.000	-0.006	-0.007	-0.008
	(-0.014 - 0.014)	(-0.015 - 0.015)	(-0.027 - 0.014)	(-0.031 - 0.017)	(-0.036 - 0.020)
Cough	-0.008	-0.010	-0.020**	-0.018	-0.012
	(-0.021 - 0.004)	(-0.025 - 0.006)	(-0.040 - -0.001)	(-0.042 - 0.007)	(-0.039 - 0.014)
Headache	0.000	0.000	-0.000	-0.000	0.002
	(-0.004 - 0.004)	(-0.005 - 0.005)	(-0.008 - 0.007)	(-0.008 - 0.008)	(-0.008 - 0.012)
Smell and Taste Disorder	0.001	0.000	0.003	-0.001	0.000
	(-0.004 - 0.006)	(-0.004 - 0.005)	(-0.003 - 0.008)	(-0.007 - 0.004)	(-0.006 - 0.006)
Panel B. Random Effect Logistic Regression Model					
High Fever	1.515	1.618*	1.498	1.554	1.938
	(0.920 - 2.497)	(0.938 - 2.792)	(0.720 - 3.118)	(0.694 - 3.481)	(0.750 - 5.008)
Sore Throat	1.002	1.016	0.888	0.885	0.910
	(0.825 - 1.217)	(0.817 - 1.263)	(0.668 - 1.180)	(0.647 - 1.211)	(0.631 - 1.311)
Cough	0.917	0.896	0.765**	0.768*	0.836
	(0.767 - 1.096)	(0.735 - 1.094)	(0.590 - 0.992)	(0.576 - 1.025)	(0.593 - 1.178)
Headache	1.072	1.106	1.060	0.954	1.117
	(0.666 - 1.724)	(0.657 - 1.860)	(0.525 - 2.142)	(0.442 - 2.060)	(0.463 - 2.696)
Smell and Taste Disorder	1.165	1.062	1.579	0.623	0.911
	(0.624 - 2.175)	(0.530 - 2.129)	(0.616 - 4.046)	(0.193 - 2.010)	(0.268 - 3.097)
Prefecture FEs for Logistic Regression	yes	yes	yes	yes	yes
Individual Level Covariates	no	yes	yes	yes	yes
Exclude Respondents far from 50 Km from the Border	no	no	yes	yes	yes
Exclude Respondents within 10 Km from the Border	no	no	no	yes	no
Exclude Respondents within 20 Km from the Border	no	no	no	no	yes
Observations	29,230	23,290	17,758	15,022	10,628

Notes: The dependent variable is a binary variable for 5 subjective symptom like SARS-CoV-2 (high fever, sore throat, cough, headache, and smell and taste disorder). Panel A summarizes the results from fixed effects linear probability regression model and Panel B summarizes the results from random effect logistic regression model. Coefficients and 95 confidence intervals are reported in Panel A and odds ratios and 95 confidence intervals are reported in Panel B. Standard errors clustered in 47 prefectures are reported in parentheses in Panel A. *** p<0.01; ** p<0.05; * p<0.1.

Table SI12: Effects on Symptoms Like SARS-CoV-2 among College Graduates

	(1)	(2)	(3)	(4)	(5)
Panel A. Fixed Effect Linear Probability Model					
High Fever	0.000	-0.001	0.001	0.002	0.003
	(-0.006 - 0.007)	(-0.009 - 0.006)	(-0.006 - 0.007)	(-0.005 - 0.009)	(-0.006 - 0.011)
Sore Throat	-0.010	-0.009	-0.014	-0.017	-0.014
	(-0.029 - 0.009)	(-0.029 - 0.011)	(-0.037 - 0.009)	(-0.045 - 0.011)	(-0.051 - 0.023)
Cough	-0.013	-0.016*	-0.032***	-0.036**	-0.030
	(-0.031 - 0.005)	(-0.036 - 0.003)	(-0.055 - -0.008)	(-0.067 - -0.006)	(-0.066 - 0.006)
Headache	-0.002	-0.003	-0.000	0.003	0.005
	(-0.010 - 0.006)	(-0.011 - 0.006)	(-0.013 - 0.013)	(-0.012 - 0.018)	(-0.013 - 0.023)
Smell and Taste Disorder	0.002	0.001	0.002	0.001	0.002
	(-0.004 - 0.008)	(-0.005 - 0.008)	(-0.003 - 0.008)	(-0.005 - 0.008)	(-0.003 - 0.008)
Panel B. Random Effect Logistic Regression Model					
High Fever	0.951	0.869	1.001	0.985	0.939
	(0.587 - 1.541)	(0.523 - 1.444)	(0.508 - 1.971)	(0.456 - 2.124)	(0.404 - 2.183)
Sore Throat	0.865	0.894	0.838	0.815	0.827
	(0.692 - 1.082)	(0.702 - 1.138)	(0.614 - 1.143)	(0.579 - 1.147)	(0.555 - 1.231)
Cough	0.842	0.815*	0.687**	0.651**	0.669**
	(0.678 - 1.048)	(0.644 - 1.031)	(0.507 - 0.930)	(0.465 - 0.911)	(0.451 - 0.991)
Headache	0.803	0.784	1.036	1.202	1.040
	(0.498 - 1.293)	(0.471 - 1.304)	(0.515 - 2.081)	(0.567 - 2.548)	(0.448 - 2.414)
Smell and Taste Disorder	1.227	1.109	1.143	1.060	1.215
	(0.640 - 2.354)	(0.562 - 2.187)	(0.456 - 2.864)	(0.389 - 2.888)	(0.391 - 3.778)
Prefecture FEs for Logistic Regression	yes	yes	yes	yes	yes
Individual Level Covariates	no	yes	yes	yes	yes
Exclude Respondents far from 50 Km from the Border	no	no	yes	yes	yes
Exclude Respondents within 10 Km from the Border	no	no	no	yes	no
Exclude Respondents within 20 Km from the Border	no	no	no	no	yes
Observations	20,017	16,581	13,427	11,630	8,452

Notes: The dependent variable is a binary variable for 5 subjective symptom like SARS-CoV-2 (high fever, sore throat, cough, headache, and smell and taste disorder). Panel A summarizes the results from fixed effects linear probability regression model and Panel B summarizes the results from random effect logistic regression model. Coefficients and 95 confidence intervals are reported in Panel A and odds ratios and 95 confidence intervals are reported in Panel B. Standard errors clustered in 47 prefectures are reported in parentheses in Panel A. *** $p<0.01$; ** $p<0.05$; * $p<0.1$.

Table SI13: Effects on Symptoms Like SARS-CoV-2 among Non College Graduates

	(1)	(2)	(3)	(4)	(5)
Panel A. Fixed Effect Linear Probability Model					
High Fever	0.005*	0.007*	0.002	0.002	0.003
	(-0.000 - 0.011)	(-0.001 - 0.014)	(-0.009 - 0.014)	(-0.012 - 0.016)	(-0.011 - 0.018)
Sore Throat	0.011	0.010	-0.004	-0.003	-0.019
	(-0.004 - 0.025)	(-0.007 - 0.027)	(-0.028 - 0.019)	(-0.030 - 0.024)	(-0.048 - 0.011)
Cough	0.005	0.007	0.003	0.005	-0.003
	(-0.009 - 0.018)	(-0.010 - 0.023)	(-0.015 - 0.022)	(-0.018 - 0.027)	(-0.033 - 0.027)
Headache	0.001	-0.001	0.001	-0.002	-0.001
	(-0.004 - 0.006)	(-0.008 - 0.006)	(-0.009 - 0.011)	(-0.013 - 0.009)	(-0.013 - 0.012)
Smell and Taste Disorder	0.001	0.000	0.001	-0.003	-0.005
	(-0.004 - 0.006)	(-0.005 - 0.005)	(-0.004 - 0.006)	(-0.009 - 0.003)	(-0.012 - 0.003)
Panel B. Random Effect Logistic Regression Model					
High Fever	1.778**	1.919**	1.412	1.577	1.732
	(1.070 - 2.955)	(1.102 - 3.343)	(0.668 - 2.983)	(0.684 - 3.637)	(0.620 - 4.842)
Sore Throat	1.190	1.194	0.957	0.991	0.834
	(0.966 - 1.467)	(0.941 - 1.515)	(0.695 - 1.319)	(0.697 - 1.409)	(0.546 - 1.272)
Cough	1.115	1.126	1.064	1.043	0.966
	(0.907 - 1.370)	(0.890 - 1.424)	(0.782 - 1.448)	(0.742 - 1.467)	(0.634 - 1.471)
Headache	1.074	1.020	1.185	1.001	1.110
	(0.661 - 1.744)	(0.598 - 1.742)	(0.584 - 2.406)	(0.447 - 2.240)	(0.407 - 3.023)
Smell and Taste Disorder	1.187	1.072	1.060	0.521	0.386
	(0.614 - 2.296)	(0.493 - 2.330)	(0.358 - 3.142)	(0.141 - 1.919)	(0.084 - 1.783)
Prefecture FEs for Logistic Regression	yes	yes	yes	yes	yes
Individual Level Covariates	no	yes	yes	yes	yes
Exclude Respondents far from 50 Km from the Border	no	no	yes	yes	yes
Exclude Respondents within 10 Km from the Border	no	no	no	yes	no
Exclude Respondents within 20 Km from the Border	no	no	no	no	yes
Observations	21,131	16,229	11,876	9,914	6,755

Notes: The dependent variable is a binary variable for 5 subjective symptom like SARS-CoV-2 (high fever, sore throat, cough, headache, and smell and taste disorder). Panel A summarizes the results from fixed effects linear probability regression model and Panel B summarizes the results from random effect logistic regression model. Coefficients and 95 confidence intervals are reported in Panel A and odds ratios and 95 confidence intervals are reported in Panel B. Standard errors clustered in 47 prefectures are reported in parentheses in Panel A. *** p<0.01; ** p<0.05; * p<0.1.

Table SI14: Effects on Symptoms Like SARS-CoV-2 among Regular Users

	(1)	(2)	(3)	(4)	(5)
Panel A. Fixed Effect Linear Probability Model					
High Fever	0.004	0.001	-0.000	0.000	0.006
	(-0.003 - 0.011)	(-0.007 - 0.008)	(-0.009 - 0.009)	(-0.010 - 0.011)	(-0.008 - 0.020)
Sore Throat	-0.002	-0.004	-0.012	-0.019	-0.005
	(-0.021 - 0.017)	(-0.023 - 0.015)	(-0.035 - 0.010)	(-0.043 - 0.005)	(-0.033 - 0.024)
Cough	-0.003	-0.006	-0.012	-0.013	-0.018
	(-0.019 - 0.014)	(-0.023 - 0.011)	(-0.034 - 0.010)	(-0.039 - 0.013)	(-0.045 - 0.010)
Headache	-0.002	-0.001	0.003	0.003	0.008
	(-0.011 - 0.007)	(-0.011 - 0.009)	(-0.014 - 0.020)	(-0.016 - 0.023)	(-0.013 - 0.029)
Smell and Taste Disorder	0.001	0.000	0.005	0.006	0.009**
	(-0.006 - 0.009)	(-0.007 - 0.007)	(-0.003 - 0.012)	(-0.002 - 0.013)	(0.001 - 0.017)
Panel B. Random Effect Logistic Regression Model					
High Fever	1.259	1.037	0.988	1.005	1.223
	(0.785 - 2.018)	(0.633 - 1.698)	(0.515 - 1.896)	(0.486 - 2.077)	(0.543 - 2.755)
Sore Throat	0.978	0.964	0.866	0.822	0.955
	(0.780 - 1.227)	(0.755 - 1.231)	(0.625 - 1.199)	(0.574 - 1.176)	(0.624 - 1.462)
Cough	1.008	0.956	0.868	0.870	0.788
	(0.806 - 1.260)	(0.751 - 1.216)	(0.629 - 1.198)	(0.609 - 1.243)	(0.514 - 1.209)
Headache	0.935	0.897	1.098	1.245	1.540
	(0.596 - 1.469)	(0.561 - 1.436)	(0.581 - 2.076)	(0.605 - 2.560)	(0.660 - 3.593)
Smell and Taste Disorder	0.981	0.947	1.542	1.719	2.226
	(0.516 - 1.863)	(0.480 - 1.870)	(0.629 - 3.781)	(0.632 - 4.675)	(0.730 - 6.787)
Prefecture FEs for Logistic Regression	yes	yes	yes	yes	yes
Individual Level Covariates	no	yes	yes	yes	yes
Exclude Respondents far from 50 Km from the Border	no	no	yes	yes	yes
Exclude Respondents within 10 Km from the Border	no	no	no	yes	no
Exclude Respondents within 20 Km from the Border	no	no	no	no	yes
Observations	17,282	14,697	11,550	9,972	7,124

Notes: The dependent variable is a binary variable for 5 subjective symptom like SARS-CoV-2 (high fever, sore throat, cough, headache, and smell and taste disorder). Panel A summarizes the results from fixed effects linear probability regression model and Panel B summarizes the results from random effect logistic regression model. Coefficients and 95 confidence intervals are reported in Panel A and odds ratios and 95 confidence intervals are reported in Panel B. Standard errors clustered in 47 prefectures are reported in parentheses in Panel A. *** $p<0.01$; ** $p<0.05$; * $p<0.1$.

Table SI15: Effects on Symptoms Like SARS-CoV-2 among Non Regular Users

	(1)	(2)	(3)	(4)	(5)
Panel A. Fixed Effect Linear Probability Model					
High Fever	0.002	0.005	0.003	0.005	0.003
	(-0.003 - 0.008)	(-0.002 - 0.011)	(-0.005 - 0.012)	(-0.006 - 0.015)	(-0.008 - 0.015)
Sore Throat	0.005	0.006	-0.005	-0.003	-0.023
	(-0.011 - 0.020)	(-0.012 - 0.024)	(-0.027 - 0.017)	(-0.028 - 0.022)	(-0.057 - 0.010)
Cough	-0.006	-0.006	-0.017	-0.020	-0.017
	(-0.018 - 0.006)	(-0.024 - 0.011)	(-0.041 - 0.007)	(-0.052 - 0.013)	(-0.050 - 0.016)
Headache	-0.001	-0.004	-0.003	-0.004	-0.005
	(-0.006 - 0.004)	(-0.010 - 0.002)	(-0.013 - 0.006)	(-0.015 - 0.007)	(-0.018 - 0.008)
Smell and Taste Disorder	0.001	0.001	-0.002	-0.006*	-0.010***
	(-0.003 - 0.005)	(-0.004 - 0.005)	(-0.008 - 0.005)	(-0.012 - 0.000)	(-0.016 - -0.003)
Panel B. Random Effect Logistic Regression Model					
High Fever	1.286	1.583	1.420	1.641	1.395
	(0.765 - 2.161)	(0.891 - 2.812)	(0.644 - 3.134)	(0.662 - 4.067)	(0.467 - 4.170)
Sore Throat	1.080	1.110	0.929	0.978	0.765
	(0.882 - 1.323)	(0.881 - 1.399)	(0.687 - 1.258)	(0.701 - 1.365)	(0.517 - 1.130)
Cough	0.945	0.936	0.830	0.773	0.799
	(0.776 - 1.152)	(0.747 - 1.173)	(0.622 - 1.107)	(0.561 - 1.065)	(0.545 - 1.172)
Headache	0.901	0.839	1.028	0.911	0.677
	(0.544 - 1.492)	(0.473 - 1.487)	(0.473 - 2.236)	(0.392 - 2.118)	(0.246 - 1.866)
Smell and Taste Disorder	1.245	1.168	0.673	0.259**	0.125**
	(0.639 - 2.423)	(0.543 - 2.510)	(0.227 - 1.992)	(0.068 - 0.991)	(0.024 - 0.657)
Prefecture FEs for Logistic Regression	yes	yes	yes	yes	yes
Individual Level Covariates	no	yes	yes	yes	yes
Exclude Respondents far from 50 Km from the Border	no	no	yes	yes	yes
Exclude Respondents within 10 Km from the Border	no	no	no	yes	no
Exclude Respondents within 20 Km from the Border	no	no	no	no	yes
Observations	23,956	18,166	13,788	11,606	8,113

Notes: The dependent variable is a binary variable for 5 subjective symptom like SARS-CoV-2 (high fever, sore throat, cough, headache, and smell and taste disorder). Panel A summarizes the results from fixed effects linear probability regression model and Panel B summarizes the results from random effect logistic regression model. Coefficients and 95 confidence intervals are reported in Panel A and odds ratios and 95 confidence intervals are reported in Panel B. Standard errors clustered in 47 prefectures are reported in parentheses in Panel A. *** p<0.01; ** p<0.05; * p<0.1.

Table SI16: Effects on Symptoms Like SARS-CoV-2 among "Not Scared"

	(1)	(2)	(3)	(4)	(5)
Panel A. Fixed Effect Linear Probability Model					
High Fever	0.004	0.002	0.003	0.006	0.007
	(-0.003 - 0.010)	(-0.006 - 0.010)	(-0.009 - 0.016)	(-0.008 - 0.021)	(-0.010 - 0.024)
Sore Throat	-0.000	-0.005	-0.006	-0.010	-0.015
	(-0.020 - 0.019)	(-0.031 - 0.021)	(-0.037 - 0.026)	(-0.045 - 0.026)	(-0.060 - 0.029)
Cough	0.005	0.003	0.005	-0.006	0.006
	(-0.015 - 0.026)	(-0.021 - 0.028)	(-0.020 - 0.030)	(-0.034 - 0.023)	(-0.027 - 0.038)
Headache	-0.001	-0.000	0.003	0.006	0.008
	(-0.010 - 0.009)	(-0.010 - 0.009)	(-0.010 - 0.017)	(-0.006 - 0.018)	(-0.006 - 0.022)
Smell and Taste Disorder	0.003	0.001	0.002	-0.001	-0.003
	(-0.005 - 0.010)	(-0.007 - 0.009)	(-0.008 - 0.012)	(-0.012 - 0.009)	(-0.017 - 0.010)
Panel B. Random Effect Logistic Regression Model					
High Fever	1.238	1.143	1.164	1.391	1.250
	(0.746 - 2.056)	(0.672 - 1.946)	(0.583 - 2.323)	(0.620 - 3.121)	(0.489 - 3.198)
Sore Throat	1.003	0.976	0.952	0.915	0.865
	(0.775 - 1.298)	(0.736 - 1.293)	(0.659 - 1.374)	(0.608 - 1.377)	(0.540 - 1.385)
Cough	1.123	1.102	1.088	0.911	1.089
	(0.875 - 1.441)	(0.839 - 1.446)	(0.763 - 1.551)	(0.610 - 1.361)	(0.680 - 1.744)
Headache	0.949	1.039	1.351	1.495	1.517
	(0.558 - 1.614)	(0.591 - 1.826)	(0.637 - 2.862)	(0.636 - 3.514)	(0.580 - 3.968)
Smell and Taste Disorder	1.266	1.233	1.334	0.960	0.844
	(0.649 - 2.469)	(0.594 - 2.557)	(0.529 - 3.368)	(0.339 - 2.722)	(0.263 - 2.708)
Prefecture FEs for Logistic Regression	yes	yes	yes	yes	yes
Individual Level Covariates	no	yes	yes	yes	yes
Exclude Respondents far from 50 Km from the Border	no	no	yes	yes	yes
Exclude Respondents within 10 Km from the Border	no	no	no	yes	no
Exclude Respondents within 20 Km from the Border	no	no	no	no	yes
Observations	14,876	12,017	9,461	8,047	5,730

Notes: The dependent variable is a binary variable for 5 subjective symptom like SARS-CoV-2 (high fever, sore throat, cough, headache, and smell and taste disorder). Panel A summarizes the results from fixed effects linear probability regression model and Panel B summarizes the results from random effect logistic regression model. Coefficients and 95 confidence intervals are reported in Panel A and odds ratios and 95 confidence intervals are reported in Panel B. Standard errors clustered in 47 prefectures are reported in parentheses in Panel A. *** p<0.01; ** p<0.05; * p<0.1.

Table SI17: Effects on Symptoms Like SARS-CoV-2 among “Scared”

	(1)	(2)	(3)	(4)	(5)
Panel A. Fixed Effect Linear Probability Model					
High Fever	0.003	0.004	0.002	0.002	0.004
	(-0.002 - 0.009)	(-0.002 - 0.010)	(-0.005 - 0.009)	(-0.006 - 0.010)	(-0.005 - 0.014)
Sore Throat	0.002	0.005	-0.010	-0.010	-0.016
	(-0.012 - 0.016)	(-0.010 - 0.019)	(-0.028 - 0.008)	(-0.032 - 0.012)	(-0.043 - 0.011)
Cough	-0.010	-0.012	-0.026**	-0.024*	-0.032**
	(-0.023 - 0.003)	(-0.027 - 0.003)	(-0.047 - -0.005)	(-0.048 - 0.001)	(-0.062 - -0.002)
Headache	-0.002	-0.005	-0.003	-0.004	-0.003
	(-0.008 - 0.003)	(-0.011 - 0.002)	(-0.015 - 0.009)	(-0.019 - 0.010)	(-0.020 - 0.014)
Smell and Taste Disorder	0.001	0.000	0.001	-0.001	0.000
	(-0.004 - 0.005)	(-0.004 - 0.005)	(-0.004 - 0.005)	(-0.006 - 0.004)	(-0.005 - 0.005)
Panel B. Random Effect Logistic Regression Model					
High Fever	1.365	1.383	1.124	1.067	1.334
	(0.845 - 2.203)	(0.821 - 2.332)	(0.534 - 2.367)	(0.479 - 2.377)	(0.531 - 3.349)
Sore Throat	1.034	1.063	0.855	0.883	0.828
	(0.858 - 1.245)	(0.863 - 1.310)	(0.648 - 1.129)	(0.652 - 1.197)	(0.576 - 1.191)
Cough	0.897	0.862	0.728**	0.757*	0.657**
	(0.746 - 1.078)	(0.701 - 1.059)	(0.556 - 0.953)	(0.563 - 1.017)	(0.458 - 0.943)
Headache	0.923	0.804	0.944	0.898	0.876
	(0.597 - 1.426)	(0.500 - 1.292)	(0.492 - 1.814)	(0.440 - 1.831)	(0.369 - 2.077)
Smell and Taste Disorder	1.080	0.976	0.983	0.676	0.821
	(0.570 - 2.044)	(0.472 - 2.017)	(0.317 - 3.047)	(0.190 - 2.401)	(0.172 - 3.910)
Prefecture FEs for Logistic Regression	yes	yes	yes	yes	yes
Individual Level Covariates	no	yes	yes	yes	yes
Exclude Respondents far from 50 Km from the Border	no	no	yes	yes	yes
Exclude Respondents within 10 Km from the Border	no	no	no	yes	no
Exclude Respondents within 20 Km from the Border	no	no	no	no	yes
Observations	26,362	20,846	15,877	13,531	9,507

Notes: The dependent variable is a binary variable for 5 subjective symptom like SARS-CoV-2 (high fever, sore throat, cough, headache, and smell and taste disorder). Panel A summarizes the results from fixed effects linear probability regression model and Panel B summarizes the results from random effect logistic regression model. Coefficients and 95 confidence intervals are reported in Panel A and odds ratios and 95 confidence intervals are reported in Panel B. Standard errors clustered in 47 prefectures are reported in parentheses in Panel A. *** p<0.01; ** p<0.05; * p<0.1.

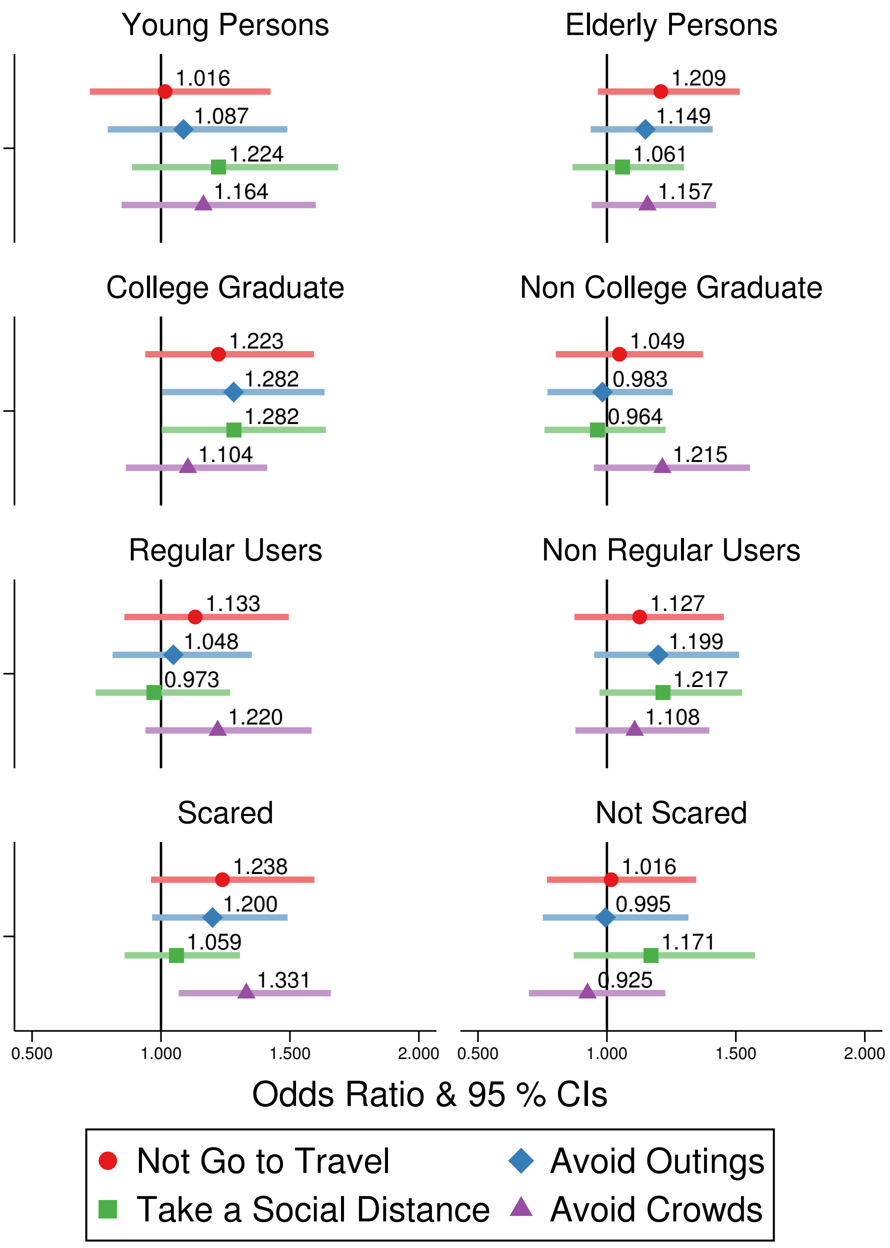

Figure SI2: Effects on Going Out Behaviors.

Note: "Young persons" represents the individuals aged 40 years old or less. "Regular Users" represents the individuals who used Japanese pubs and bars before the pandemic. "Scared" and "Not scared" represent the individuals who reported that they were scared (not scared) at SARS-CoV-2 in the first wave.
